# Triglyceride and Glucose Index and Sex Differences in Relation to Major Adverse Cardiovascular Events in Hypertensive Patients Without Diabetes

**DOI:** 10.3389/fendo.2021.761397

**Published:** 2021-11-05

**Authors:** Kun Yang, Wenxian Liu

**Affiliations:** Cardiac Care Unit, Department of Cardiology, Beijing Anzhen Hospital, Capital Medical University, Beijing Institute of Heart Lung and Blood Vessel Diseases, Beijing, China

**Keywords:** triglyceride and glucose index (TyG index), major adverse cardiovascular events (MACEs), sex differences, Systolic Blood Pressure Intervention Trial (SPRINT), hypertension

## Abstract

**Introduction:**

Studies from recent decades have suggested that women have a lower risk of cardiovascular disease than men due to their characteristics, but hyperglycemia and hyperinsulinemia caused by IR (insulin resistance) might reverse this gender-protective effect. This study examined whether there were sex differences in the relationship between IR [evaluated by triglyceride and glucose index (TyG index)] and major adverse cardiovascular events (MACEs) in hypertensive patients without diabetes.

**Methods:**

This was a *post-hoc* analysis of the Systolic Blood Pressure Intervention Trial (SPRINT). We explored the relationship between TyG index and MACEs by multivariate Cox proportional hazard regressions and two-piecewise linear regression models. The primary endpoint was MACEs, same as SPRINT, defined as a composite of myocardial infarction, stroke, heart failure, and/or death from cardiovascular causes. We used multiple adjustment models for all regressions.

**Results:**

A total of 9,323 patients from the SPRINT were included in our analysis. TyG index was significantly related to the risk of MACEs in every adjusted model. Each 1 unit increase in TyG index increased the risk of MACEs in total participants (HR, 1.40; 95% CI, 1.20–1.64; P<0.01) and men (HR, 1.42; 95% CI, 1.18–1.71; P=0.02). However, TyG index was not associated with MACEs among female hypertensive patients (HR, 1.33; 95% CI, 0.97–1.82; P=0.0776). There was no interaction between the sex and TyG index (P for interaction= 0.73). We also used the two-stage linear regression model and did not find any threshold effect. There was no significant interaction in other confounders.

**Conclusion:**

We found the TyG index was associated with MACEs in the hypertensive patients, and there was no gender difference between the TyG index and MACEs.

## Background

Insulin resistance (IR) is a key metabolic abnormality leading to the development of T2DM (1), and recent studies have also found that it could independently predict the development of cardiovascular disease (CVD) and chronic kidney disease (CKD) (2). Hyperinsulinemic-Euglycemic Clamp (HIEC), as the gold standard for the diagnosis of insulin resistance, is very difficult to be carried out in clinical work due to its difficulty of operation and equipment requirements (3). Triglyceride and glucose index (TyG index) was one of the alternative methods for evaluating insulin resistance (4) and was proved to be consistent with the HIEC in several studies (5–7). Therefore, as the accessibility of its measurement indicators (fasting plasma glucose and fasting triglyceride levels), TyG was used in numerous cardiovascular studies as an alternative to insulin resistance (8–12).

The lipotoxicity and glucotoxicity of the IR were the main factors in the development of cardiovascular diseases. Moreover, previous studies found an independent relevance between TyG and the major adverse cardiovascular events (MACEs), including acute coronary syndrome (8–10), stroke (11), and CVD death (12). However, this correlation might be different in both genders, due to their differences in physiology, cultural behavior, and environmental factors (13–15). In recent decades scientific literature has indicated that women have a lower risk of cardiovascular events due to their characteristics than men. However, the protection in female patients might be reverted with IR-related disorders due to the hyperglycemia and hyperinsulinemia caused by IR (16, 17).

The population included in the previous studies is the general population (12). Few studies included a large proportion of the elderly, and the elderly tended to have more cardiovascular risk factors. Moreover, previous studies did not distinguish between diabetic and non-diabetic patients. Previous studies from different regions also found that there might be gender differences in the correlation between the TyG index and MACEs (12, 18, 19). To better study these issues, we used the data from the Systolic Blood Pressure Intervention Trial (SPRINT) (20) to evaluate the relationship between TyG index and MACEs in a hypertension population and further explore the correlation between gender differences.

## Method

We performed a *post-hoc* analysis of the SPRINT. The limited dataset was obtained from the National Institutes of Health Biologic Specimen and Data Repository Information Coordinating Center (https://biolincc.nhlbi.nih.gov/studies/sprint/).

### Study Population

SPRINT was a randomized, controlled trial conducted at 102 clinical sites in the United States. The rationale, design, and main results of SPRINT have been previously published (20, 21). Briefly, SPRINT was designed to test whether the intensive management of systolic BP to <120 mmHg reduces cardiovascular disease events compared with standard BP management (<140 mmHg). The recruited participants were between the ages of 50 and 75 and had at least one of the following: presence of clinical or subclinical cardiovascular disease other than stroke; chronic kidney disease (defined as eGFR 20–59 ml/min/1.73 m2); Framingham risk score for 10-year CVD risk ≥15% based on laboratory work done in the last 12 months; or if patients were aged 75 years or older. Because the blood pressure trial of ACCORD study did not come to a good conclusion within the diabetic patients (22). Exclusion criteria were that patients had type 2 diabetes, prior stroke, and standing systolic BP <110 mmHg at the screening visit. The SPRINT showed that intensive blood pressure management significantly reduced cardiovascular mortality and all-cause mortality compared to standard management.

### Exposure Variables

TyG index was defined as TyG=Ln [fasting triglycerides (mg/dl) × fasting glucose (mg/dl)/2]. We used the baseline fasting triglycerides and fasting glucose to calculate the TyG index (7, 23). We divided the population into three groups according to the size of the TyG index. The first group was the reference.

The primary endpoint of our study was major cardiovascular adverse events (MACEs), defined as a composite of myocardial infarction, stroke, heart failure, and/or death from cardiovascular causes. The definitions of MI, stroke, heart failure, and outcomes were the same as SPRINT and presented elsewhere. The outcomes were adjudicated.

### Statistical Analysis

The baseline characteristics and outcomes of patients were expressed as frequencies and percentages for categorical variables. Means and standard deviations (SDs) or median and interquartile ranges were used for continuous variables, depending on whether datasets were normally distributed (assessed using normal Q–Q plots). We used chi-square analysis to compare categorical variables. We used analysis of variance or the Mann–Whitney U test to compare continuous variables in accordance with the distribution type.

The adjusted variables in this study were selected based on their clinical importance. Three multivariate Cox proportional hazard regressions were constructed to estimate the association of the baseline TyG index with the risk of MACEs by calculating the hazard ratio (HR) and 95% confidence interval (CI). The validity of the proportionality assumption was verified by scaled Schoenfeld residuals. Model 1 was an unadjusted model. Model 2 was adjusted for age, treatment arm, and ethnicity. Model 3 was further adjusted for age, treatment arm, ethnicity, baseline body mass index, smoking status, chronic kidney disease (CKD) subgroup, cardiovascular disease (CVD) subgroup, number of antihypertensive agents, aspirin used, and statin used at baseline.

To account for the TyG index as a continuous variable, we constructed a Cox proportional hazards regression model (Model 3). The TyG index was used to calculate the HR for outcomes. Then restricted cubic spline models with four knots at the fifth, 35th, 65th, and 95th percentiles were built to detect any non-linear relationship between TyG index and mortality. We used two-piecewise linear regression models to elucidate how the associations differed by the threshold point. The threshold value was estimated by trying all possible values and choosing the threshold point with the highest likelihood. A logarithmic likelihood ratio test was employed to compare the differences in associations when using one-line linear regression models *vs.* two-piecewise linear regression models.

We performed the interaction and stratified analyses by sex, treatment arm, age (<75 years and ≥75 years), systolic blood pressure tertile (≤132 mmHg, 132–145 mmHg, and ≥145 mmHg), Framingham 10-y cardiovascular disease risk score (≤15%, >15%), smoking status, CVD subgroup, CKD subgroup, Black race, aspirin use, and statin use.

All analyses were performed using statistical software packages R (The R Foundation; http://www.R-project.org) and EmpowerStats (X&Y Solutions, Inc., Boston, MA, USA; http://www.empowerstats.com). P values <0.05 (two-sided) were considered statistically significant.

## Results

### Baseline Characteristics of Included Hypertension Patients

Among the total 9,323 patients with hypertension from the SPRINT trial, there are 38 patients whose TyG index cannot be calculated. The median follow-up was 3.26 years. After follow-up, 155 (4.99%) MACEs occurred in the low TyG group, 196 (6.30%) in the middle group, and 210 (6.76%) in the high TyG group. Low TyG group patients had higher HDL-C levels and GFR. High TyG group patients had a higher Framingham 10-y cardiovascular disease risk score. **Table 1** provides the detailed baseline characteristics of the patients with hypertension included in the study population.

**Table 1:** Baseline characteristics and crude end points of the study participants.

	*TYG Tertile*			
	Low	Middle	High	*P*-Value
N	3,104	3,111	3,108	
TYG Scores	8.02 ± 0.23	8.55 ± 0.13	9.19 ± 0.35	<0.01
Intensive treatment	1,554 (50.06%)	1,580 (50.79%)	1,528 (49.16%)	0.44
BMI	28.58 ± 5.84	30.01 ± 5.86	30.96 ± 5.34	<0.01
Age, y				
Overall	69.15 ± 9.65	68.26 ± 9.35	66.31 ± 9.03	<0.01
≥75 y, n (%)	1,037 (33.41%)	929 (29.86%)	659 (21.20%)	<0.01
Race, n (%)				<0.01
Non-Hispanic Black	1,228 (39.56%)	911 (29.28%)	646 (20.79%)	
Hispanic	210 (6.77%)	339 (10.90%)	429 (13.80%)	
Other	54 (1.74%)	48 (1.54%)	72 (2.32%)	
Non-Hispanic White	1,612 (51.93%)	1,813 (58.28%)	1,961 (63.10%)	
Black Race, n (%)	1,281 (41.27%)	955 (30.70%)	694 (22.33%)	<0.01
Baseline blood pressure, mmHg				
Systolic	140.13 ± 15.63	139.60 ± 15.76	139.27 ± 15.34	0.90
Diastolic	77.47 ± 12.26	77.80 ± 11.62	79.11 ± 11.88	<0.01
Distribution of systolic blood pressure, n (%)				0.16
≤132 mmHg	999 (32.18%)	1,070 (34.39%)	1,055 (33.94%)	
>132 to <145 mmHg	1,002 (32.28%)	994 (31.95%)	1,030 (33.14%)	
≥145 mmHg	1,103 (35.53%)	1,047 (33.65%)	1,023 (32.92%)	
Serum creatinine, mg/dl	1.06 ± 0.33	1.07 ± 0.34	1.09 ± 0.35	0.03
Estimated GFR, ml* min^−1^*1.73 m^−2^	73.04 ± 20.79	71.32 ± 20.49	70.88 ± 20.44	<0.01
Fasting HDL cholesterol, mg/dl	60.92 ± 15.64	52.19 ± 12.18	45.52 ± 10.75	<0.01
Fasting LDL cholesterol, mg/dl	107.00 ± 31.53	113.91 ± 34.58	116.36 ± 38.31	<0.01
Fasting total cholesterol, mg/dl	181.38 ± 37.67	187.61 ± 38.91	201.35 ± 44.08	<0.01
Fasting total triglycerides, mg/dl	67.33 ± 14.77	107.56 ± 17.30	202.86 ± 119.89	<0.01
Fasting glucose, mg/dl	93.36 ± 9.93	98.25 ± 11.15	104.83 ± 16.16	<0.01
Statin use, n (%)	1,278 (41.52%)	1,387 (44.87%)	1,381 (44.65%)	0.01
Aspirin use, n (%)	1,578 (50.97%)	1,625 (52.32%)	1,544 (49.77%)	0.13
Smoking status, n (%)				0.51
Never smoked	1,414 (45.55%)	1,358 (43.65%)	1,339 (43.08%)	
Former smoker	1,286 (41.43%)	1,339 (43.04%)	1,338 (43.05%)	
Current smoker	401 (12.92%)	409 (13.15%)	428 (13.77%)	
Framingham 10-y cardiovascular disease risk score, %	17.73 ± 9.33	20.02 ± 10.79	22.52 ± 11.76	<0.01
No. of antihypertensive agents	1.80 ± 1.04	1.83 ± 1.04	1.87 ± 1.04	0.06
Not using antihypertensive agents, n (%)	309 (9.95%)	282 (9.06%)	289 (9.30%)	0.46
MACEs	155 (4.99%)	196 (6.30%)	210 (6.76%)	0.01

GFR, glomerular filtration rate; LDL, low-density lipoprotein; HDL, high-density lipoprotein.

Plus–minus values are means ± SD. To convert the values for creatinine to micromoles per liter, multiply by 88.4. To convert the values for cholesterol to millimoles per liter, multiply by 0.02586. To convert the values for triglycerides to millimoles per liter, multiply by 0.01129. To convert the values for glucose to millimoles per liter, multiply by 0.05551.

Race and ethnic group were self-reported.

Black race includes Hispanic black and black as part of a multiracial identification.

The body mass index is the weight in kilograms divided by the square of the height in meters.

### Tertiles of TyG Index and MACEs

The association between the TyG index and MACEs in patients with hypertension is presented in **Table 2**. No matter in which model, the TyG index was significantly related to the risk of MACEs. In Model 3, the third tertile has the highest risk of MACEs (HR, 1.45; 95% CI, 1.17–1.81; P<0.01). When the TyG index grouping was regarded as a continuous variable, this trend did not change (HR, 1.20; 95% CI, 1.08–1.34; P<0.01). A similar trend was observed in male patients. Although there were similar trends among female patients, the statistical difference was not significant.

**Table 2:** TyG index tertile and MACEs.

TyG scores tertile	Hazard ratio (95% CI) P-value		
	Model 1	Model 2	Model 3
Male			
1	Ref	Ref	Ref
2	1.24 (0.96, 1.60) 0.10	1.32 (1.02, 1.71) 0.03	1.27 (0.98, 1.65) 0.07
3	1.34 (1.05, 1.72) 0.02	1.62 (1.26, 2.09) <0.01	1.46 (1.13, 1.90) <0.01
TyG index tertiles as a continuous variable	1.15 (1.02, 1.30) 0.02	1.27 (1.12, 1.44) <0.01	1.20 (1.06, 1.37) <0.01
Female			
1	Ref	Ref	Ref
2	1.27 (0.87, 1.84) 0.21	1.34 (0.92, 1.95) 0.12	1.32 (0.90, 1.92) 0.16
3	1.26 (0.86, 1.85) 0.24	1.45 (0.97, 2.15) 0.07	1.41 (0.94, 2.10) 0.10
TyG index tertiles as a continuous variable	1.12 (0.93, 1.35) 0.23	1.20 (0.99, 1.46) 0.06	1.18 (0.97, 1.44) 0.09
All participants			
1	Ref	Ref	Ref
2	1.25 (1.01, 1.54) 0.04	1.33 (1.07, 1.64) <0.01	1.29 (1.04, 1.60) 0.02
3	1.32 (1.07, 1.63) <0.01	1.56 (1.26, 1.94) <0.01	1.45 (1.17, 1.81) <0.01
TyG index tertiles as a continuous variable	1.14 (1.03, 1.27) <0.01	1.25 (1.12, 1.39) <0.01	1.20 (1.08, 1.34) <0.01

Model 1, unadjusted; model 2, adjusted for age, treatment arm, and ethnicity; model 3, full adjusted model, adjusted for age, treatment arm, ethnicity, baseline body mass index, chronic kidney disease (CKD) subgroup, cardiovascular disease (CVD) subgroup, aspirin used, and statin used. Ref, reference.

### TyG Index as a Continuous Variable and MACEs

**Figure 1:**
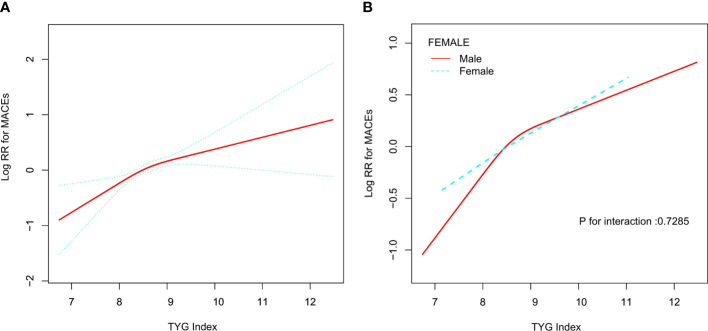
Relationship between Tyg index and MACEs. **(A)** Relationship between Tyg index and MACEs in all patients. The red line is the trend line and the blue line is the 95% confidence interval. **(B)** Relationship between Tyg index and MACEs grouped by sex. Male: red line; Female: blue line.

As shown in **Table 3**, when we used the TyG index as a continuous covariate, each 1 unit increase in TyG index increased the risk of MACEs in total participants (HR, 1.40; 95% CI, 1.20–1.64; P<0.01) and men (HR, 1.42; 95% CI, 1.18–1.71; P<0.01). However, the TyG index was not associated with MACEs among female hypertensive patients (HR, 1.33; 95% CI, 0.97–1.82; P=0.08). Restricted cubic splines were used to flexibly model and visualize the relationship between the TyG index and MACEs. With the increase of the TyG index, the risk of MACEs increased. When the TyG index was close to nine, the trend of increasing the risk of MACEs slowed down (**Figure 1**). There was no interaction between the sex and TyG index (P for interaction= 0.73).

**Table 3:** Results of two-piecewise linear-regression model.

	Male	Female	Total
One linear-regression model	1.42 (1.18, 1.71) P<0.01	1.33 (0.97, 1.82) P=0.08	1.40 (1.20, 1.64) P<0.01
Inflection point (K)	8.72	7.76	8.71
<K Effect size β (95% CI)	1.84 (1.26, 2.69) P<0.01	30.84 (0.04, 22031.46) 0.3065 P=0. 31	1.66 (1.21, 2.27) P<0.01
>K Effect size β (95% CI)	1.14 (0.81, 1.61) P=0.45	1.26 (0.90, 1.75) P=0.18	1.20 (0.89, 1.62) P=0. 22
Log likelihood ratio test	0.118	0.274	0.214

Two-piecewise linear-regression model was used to calculate the threshold effect of the TyG index. If the log likelihood ratio test >0.05, it means the two-piecewise linear regression model is not superior to the single-line linear regression model.

Next, we used the two-stage linear regression model to calculate the threshold effect. **Table 3** shows the results of the two-stage linear regression model. The inflection point was 8.71 in all participants; on the left inflection point, the effect size, 95% CI, and P value were 1.66, 1.21–2.27, and <0.01, respectively; on the right inflection point, HR, 1.20; 95% CI, 0.89–1.62; P=0.22. However, the log likelihood ratio test was 0.21. This means that the two-stage linear regression model was not better than the one-line linear regression models.

### Interaction and Sensitivity Analyses

**Figure 2:**
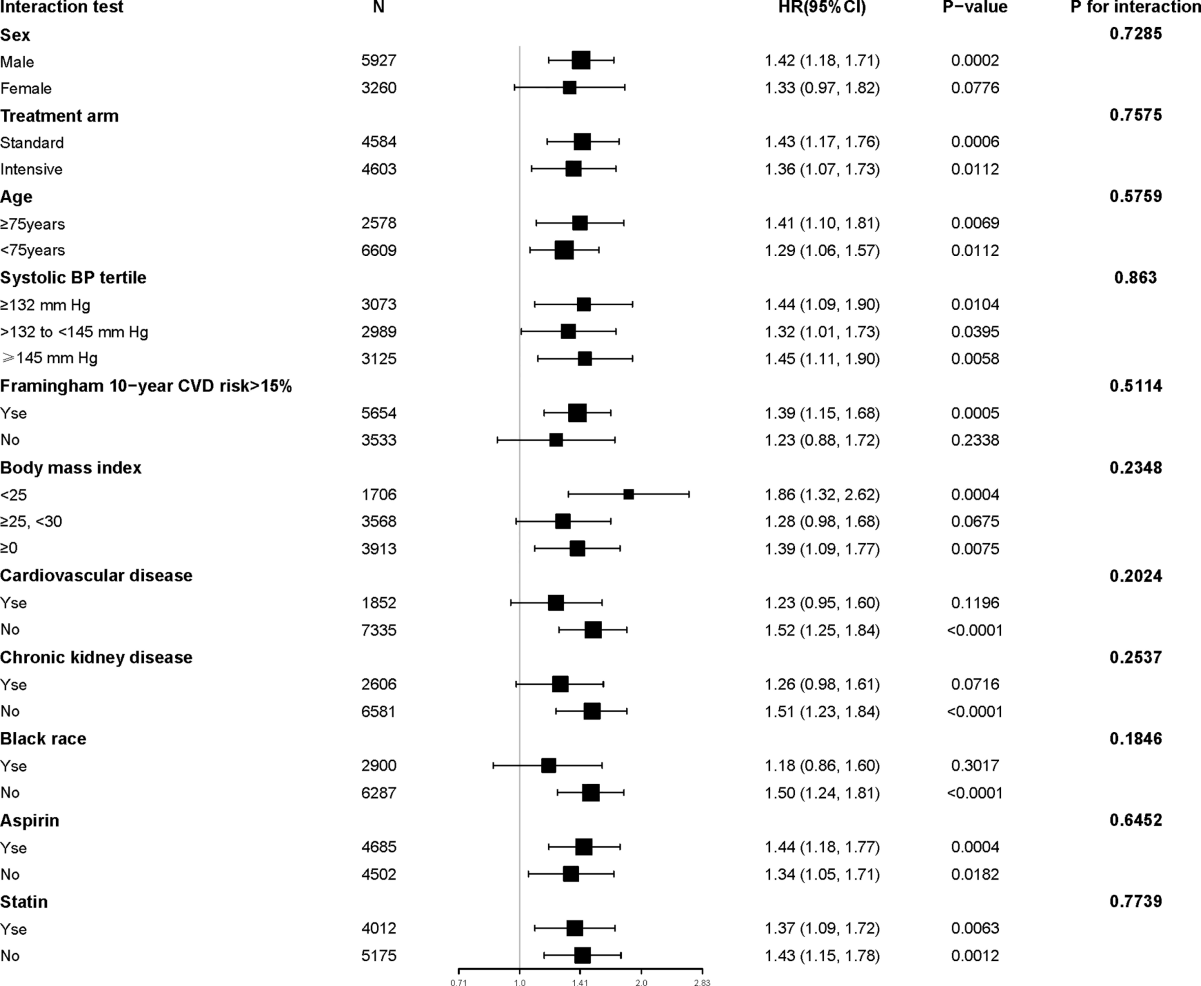
Subgroup analysis of the relationship between Tyg index and MACEs.

The results of the interaction and stratified analyses are presented in **Figure 2**. Generally, the TyG index was significantly associated with the risk of MACEs across various subgroups. There was no significant interaction in the confounders.

## Discussion

In this *post hoc* analysis, we observed that a high baseline TyG index was associated with the risk of MACEs in old hypertensive patients. And there was no significant interaction in sex and the TyG index.

TyG index, which is calculated from fasting triglyceride and blood glucose, is a reliable and surrogate biomarker to assess insulin resistance. The accuracy of TyG index in the diagnosis of insulin resistance was consistent with that of HIEC and HOMA-IR (4). A recent meta-analysis involving 5,731,294 participants reported that a higher TyG index might be independently associated with a higher incidence of atherosclerotic cardiovascular diseases (ASCVDs), CAD, and stroke in people without ASCVDs at baseline (24). To the best of our knowledge, this study is the first study to evaluate the relationship between the TyG index and adverse outcomes within the population of hypertensive patients without diabetes. These patients tended to have a higher risk of ASCVDs. A similar previous study focusing on the general population reached a similar conclusion to ours. The average age of the population we included in the study was much older than them, and the value of the inflection point we obtained was smaller than theirs. This explains to a certain extent that as patients age and cardiovascular risk factors increase, the risk of adverse events caused by an increase in the TyG index is higher (12). The association between the TyG index and the risk of MACEs should be interpreted as insulin resistance reflected by the TyG index. Insulin resistance is a condition where the body tissues became resistant to insulin, resulting in a disorder of lipid and glucose metabolism (25). The main characteristic of dyslipidemia caused by insulin resistance is the lipid triad, including hypertriglyceridemia, low HDL cholesterol, and small and dense LDL. In a normal physiological state, insulin could degrade apoB and reduce VLDL synthesis through activation of PI3K. However, in the insulin resistance state, this degradation is inhibited, resulting in increased VLDL synthesis (26). Hyperglycemia caused by insulin resistance could induce long-term epigenetic modifications of the NF-κB promoter, leading to mitochondrial dysfunction and endoplasmic reticulum stress (27). This could lead to an increase in reactive oxygen species and inflammatory factors, which could impair endogenous nitric oxide release and cause endothelial dysfunction (28). These insulin resistance states could promote the development of cardiovascular and cerebrovascular diseases (29, 30). Insulin resistance was also significantly associated with coronary artery plaque vulnerability, leading to the occurrence of acute coronary syndrome (31, 32). Therefore, these metabolic disorders promoted endothelial dysfunction, cardiovascular remodeling, oxidative stress, inflammatory factors release that exacerbated elevated blood pressure and artery stiffness, all of which were major risk factors for cardiovascular diseases (30, 33).

Previous studies have found gender differences in the relationship between the TyG index and the risk of major adverse cardiovascular events. The Kailuan cohort study found that the TyG index had an interaction with gender in predicting myocardial infarction, and the TyG index had a better predictive efficiency in women (34). Just like other traditional cardiovascular risk factors, there were gender differences in the relationship between the TyG index and cardiovascular diseases. The sex difference in the association between traditional cardiovascular risk factors could be explained by increased insulin resistance in menopausal women with reduced estrogen levels, which lead to a higher risk of cardiovascular diseases (35). However, our study found no interaction between the TyG index and gender in predicting MACEs. The reason for the inconsistency with the Kailuan cohort study may be the difference in study population size and the short follow-up time. The average age of the population included in our study was significantly greater than in that study. This might weaken the gender differences caused by menopausal women.

Despite the above strengths and potential clinical implications, this research had some limitations that should be considered when interpreting the results. First, this was a *post-hoc* analysis, and the original study was not designed to examine the relationship between the TyG index and MACEs. Second, the study had a short follow-up time, and this limited the application of the results of this study.

## Conclusion

In this *post-hoc* analysis using data from the SPRINT, we found that the TyG index was associated with MACEs in the hypertensive patients, and there was no gender difference between the TyG index and MACEs.

## Data Availability Statement

Publicly available datasets were analyzed in this study. This data can be found here: https://biolincc.nhlbi.nih.gov/studies/sprint/.

## Author Contributions

KY wrote the paper. KY applied for the database and made statistical analysis. WL was responsible for the revision of the paper. Both authors contributed to the article and approved the submitted version.

## Conflict of Interest

The authors declare that the research was conducted in the absence of any commercial or financial relationships that could be construed as a potential conflict of interest.

## Publisher’s Note

All claims expressed in this article are solely those of the authors and do not necessarily represent those of their affiliated organizations, or those of the publisher, the editors and the reviewers. Any product that may be evaluated in this article, or claim that may be made by its manufacturer, is not guaranteed or endorsed by the publisher.
